# Preparation of nanocellulose from bagasse and its application in chitosan composite films

**DOI:** 10.1098/rsos.242253

**Published:** 2025-07-23

**Authors:** Li Wu, Shengli Gao, Hao Cheng, Chengjie Wu, Shuai Zhang, Ruiping Gao, Xiaohua Zhu

**Affiliations:** ^1^ Chongqing Technology and Business University, Chongqing, People’s Republic of China; ^2^ Guangxi University of Science and Technology, Liuzhou, Guangxi, People’s Republic of China; ^3^ Zhaoqing University, Zhaoqing, People’s Republic of China; ^4^ Key Laboratory Marine Biological Waste and Comprehensive Utilization of Guangdong Province, Zhanjiang, People’s Republic of China

**Keywords:** nanocellulose, chitosan, composite membrane, barrier properties

## Abstract

Cellulose nanocrystals (CNC) are promising for preparing composite materials for food packaging and preservation; however, few studies have utilized nanocellulose from sugarcane bagasse for developing composite membranes. In this study, CNC from bagasse was prepared, CNC/chitosan (CS)/glycerol composite membranes were obtained by the mixing and extensional flow method, and the formulations were optimized using single-factor and orthogonal tests to investigate the effects of CNC, CS and glycerol on the mechanical properties, barrier properties, structure and thermal stability of the composite films. The composite film prepared showed better performance at CNC, CS and glycerol concentrations of 1, 1.5 and 0.75%, respectively. Compared with those of the CS membrane, the barrier performance was improved (*p* < 0.05). The morphology of CNC and CNC/CS composite films was observed by scanning electron microscopy. The structure was characterized by Fourier transform infrared spectroscopy and X-ray diffraction, and thermal stability was analysed by thermogravimetric analysis. Hydrogen bonding between nanocellulose and CS enhanced the cohesion of the composite film. The newly prepared CNC/CS composite film showed good mechanical properties, barrier properties and stability. It has potential applications in the food packaging industry to extend the shelf life of food and is energy-saving and environmentally friendly.

## 1. Introduction

Food waste is a major issue worldwide. Fruits, vegetables and meat are prone to spoilage due to their short shelf life and harsh storage conditions and constitute a large percentage of food waste. The development of environmentally friendly preservation materials to ensure food safety and extend food shelf life is a pertinent technological challenge. Recent research has focused on materializing renewable biological resources, which can mitigate CO_2_ emissions, alleviate environmental pollution and foster sustainable development [1–3]. Cellulose, a long-chain linear polysaccharide with the formula (C_6_H_10_O_5_)n comprising glucose monomers through β-(1,4)-glycosidic bonds, is the most abundant natural polymer on earth, with a global annual production of approximately 7.5 × 10^10^−10.0 × 10^10^ t [4]. It is known as the ‘green, inexhaustible asset’. Nanocrystalline cellulose is a nanoscale (≤100 nm) cellulose material with excellent mechanical strength, biocompatibility and renewability, making it promising for the development of new green composites [5]. Nanocellulose is generally prepared from natural cellulose, including cellulose nanofibrils, bacterial cellulose and cellulose nanocrystals (CNC) [6]. Reactive groups and the structure of nanocellulose facilitate binding to other compounds; accordingly, it is often used to prepare composite and coated film materials, with applications in several fields including smart films, food packaging, food preservation and biomedicine [7–10].

CNC is mainly an amorphous short-range rod or coniferous crystalline material. Compared with those of cellulose nanofibrils and bacterial cellulose, CNC has a lower aspect ratio (length <500 nm, diameter 5−80 nm) but a higher degree of crystallinity (50−90%), endowing it with good mechanical properties. It can be used to stabilize nanoemulsions and improve the mechanical strength and mechanical properties of composite membranes [11,12]. The use of CNC to prepare composite membranes is beneficial with respect to mechanical strength and mechanical properties. Abundant hydroxyl groups on the CNC surface can improve water-retention properties, aiding better food storage. Using a central combinatorial design approach, Oliveira *et al.* [13] found that incorporating CNC, maleic anhydride and *Lactococcus lactis* nisZ improved the antimicrobial effects and mechanical features of corn starch/polyvinyl alcohol composite membranes but weakened the barrier function. Noorbakhsh-Soltani *et al.* [14] added CNC to nanocomposite films with starch/chitosan (CS) and gelatin/CS as substrates; using response surface methodology, they found that Young’s modulus, tensile strength (TS), elongation at break (EB) and food preservation capacity of the two composite films improved when nanocrystalline cellulose was added at 8%. Fakouri *et al.* [15] found that a CNC/gelatin film-forming solution could prolong the shelf life of strawberries from 2−3 to 8 days.

Sugarcane is among the main agricultural products in tropical and subtropical regions; 80% of sugar on the global market comes from sugarcane. Bagasse is a potential raw material for preparing green cellulose-derived materials [16]. For every 1000 t of sugarcanes processed, 270 t of bagasse is produced [17]; the bagasse content in sugarcane is 32−45%. Latif *et al.* [16] extracted cellulose from bagasse and corn cob waste and utilized it in a mixed form to prepare bioplastic film. After 12 days of incubation of sherbet berries, complete spoilage was identified in the control group compared to those covered with the bioplastic film. Oliveira *et al.* [18] obtained CNC with a high aspect ratio and crystallinity index from bagasse cellulose by sulfuric acid hydrolysis. Aguiar *et al.* [19] extracted CNC with a high crystallinity index and thermal stability from bagasse cellulose using enzymatic digestion. However, relatively few studies have evaluated the application of bagasse nanocellulose in composite membranes. CS is a natural polysaccharide with excellent film-forming properties and antimicrobial effects; it has been the focus of extensive research and has wide applications in film-coating preservation [20,21]. However, intramolecular and intermolecular network gaps on CS glucosidic chains lead to poor water vapour barrier properties and high brittleness of CS films [22]. Therefore, preservation effects are limited and the structure can be easily damaged. In this study, CNC was extracted from bagasse for the preparation of CS–CNC composite membranes with improved mechanical and barrier properties, providing a basis for new cellulose-based food packaging materials.

## 2. Results and discussion

### 2.1. Bagasse nanocellulose

#### 2.1.1. Morphology and yield of nanocellulose

**Figure 1.**
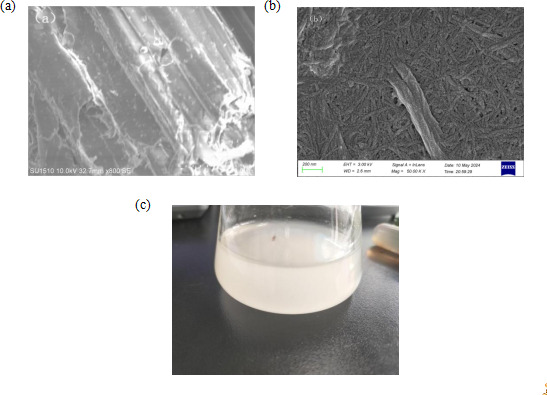
SEM morphology of (a) cellulose and (b) CNC; morphology of CNC solution (c).

Figure 1a shows a representative scanning electron microscope（SEM）image of bagasse cellulose. The cellulose after alkaline extraction comprised multiple fibres affixed together. Short rods were observed after acidic nanosizing treatment, as shown in figure 1b, indicating that the cellulose underwent a significant morphological change. The prepared nanocellulose solution was milky white in colour, as shown in figure 1c. The nanocellulose yield, calculated from equation (4.1), was 31.3 ± 1.25%.

#### 2.1.2. Particle size analysis

The mean size was 230 nm, and 44.94 nm fibres accounted for 77.2%. The PDI (representing the heterogeneity of particle sizes) was 0.396, indicating that the nanocellulose particles were concentrated and the variation in particle size was low. Therefore, the distribution of nanocellulose particles was relatively even in the nanocellulose solution. Other relatively larger cellulose particles can form by the incomplete hydrolysis of cellulose or the recombination of nanocellulose to form large particle compounds through forces including hydrogen bonds.

### 2.2. CNC/CS composite film process optimization

#### 2.2.1. Influence of the CNC concentration on the performance of the composite film

As shown in table 1, the thickness of the composite film decreased and then increased as the CNC content increased (*p* < 0.05). TS and EB tended to increase initially and then decrease (*p* < 0.05), with the highest values at a concentration of 1% (25.45 MPa and 45.41%, respectively). These findings suggest that a CNC concentration of 1% can improve the mechanical properties of composite films. This can be explained by the large specific surface area of CNC, which can form a more compact network structure with CS molecules through hydrogen bonding and van der Waals forces [23], thereby improving the resistance of the composite membrane to external forces and enhancing membrane flexibility. However, an excessive amount of cellulose can cause aggregation [24], thereby decreasing the uniformity of the film. In the area of aggregation, stress can accumulate, decreasing the flexibility of the membrane.

**Table 1.** Effect of CNC/CS/glycerol concentration on the properties of the CNC/CS composite films.

material	concentration (%）	thickness (mm)^A^	TS (MPa)^B^	EB (%)^C^
CNC	0.50	0.067 ± 0.0006^b^	18.61 ± 1.63^c^	33.78 ± 1.46^d^
	0.75	0.062 ± 0.0010^c^	23.35 ± 0.96^b^	37.94 ± 1.57^c^
	1.00	0.056 ± 0.0015^d^	25.45 ± 0.42^a^	45.41 ± 1.93^a^
	1.25	0.066 ± 0.0006^b^	24.87 ± 0.56^ab^	42.72 ± 0.54^ab^
	1.50	0.074 ± 0.0015^a^	23.71 ± 0.57^b^	41.09 ± 1.21^b^
CS	0.50	0.052 ± 0.0026^d^	24.72 ± 1.09^ab^	39.38 ± 0.82^c^
	1.00	0.058 ± 0.0006^c^	25.41 ± 0.53^ab^	42.81 ± 0.87^b^
	1.50	0.057 ± 0.0020^c^	25.91 ± 0.66^a^	46.76 ± 1.17^a^
	2.00	0.072 ± 0.0036^b^	23.84 ± 0.91^ab^	45.83 ± 1.71^a^
	2.50	0.083 ± 0.0025^a^	23.49 ± 1.5^ab^	43.29 ± 0.83^b^
glycerol	0.50	0.058 ± 0.0025^c^	30.99 ± 1.91^a^	42.88 ± 1.02^d^
	0.75	0.061 ± 0.0021^c^	27.07 ± 1.79^a^	45.89 ± 0.82^c^
	1.00	0.076 ± 0.0021^b^	25.73 ± 0.50^b^	47.74 ± 1.03^bc^
	1.25	0.083 ± 0.0010^a^	22.13 ± 1.25^c^	48.76 ± 0.95^ab^
	1.50	0.085 ± 0.0025^a^	20.65 ± 0.53^c^	50.33 ± 0.85^a^

^A^ a–d indicate significant differences (*p* < 0.05) within column thickness (mm).

^B^ a–c indicate significant differences (*p* < 0.05) within column tensile strength (MPa).

^C^ a–d indicate significant differences (*p* < 0.05) within column elongation (%).

#### 2.2.2. Influence of the CS concentration on composite film performance

As shown in table 1, the thickness of the composite film increased as the CS content increased (*p* < 0.05). TS and EB showed an initial increase, followed by a decreasing trend (*p* < 0.05). TS did not show significant differences in CS concentration (*p >* 0.05). However, EB showed significant differences among groups (*p <* 0.05); at 1.5% CS, EB was highest (46.76%), TS was 25.91 MPa and the composite film showed the best mechanical properties. As a polymer, chitosan shows hydrogen bonding with nanocellulose, enhancing the network structure of the composite film and ultimately increasing the TS and EB. However, as the concentration increased, the high molecular weight of chitosan caused the network microstructure to break. The six-member ring structure in the main chain had difficulty rotating inward, and the effects of strong chemical intramolecular and intermolecular bonds increased rigidity and lowered the EB values [25].

#### 2.2.3. Influence of glycerol concentration on the performance of the composite film

As shown in table 1, the thickness of the composite film increased (*p* < 0.05), TS decreased (*p* < 0.05) and EB increased (*p* < 0.05) as the glycerol content increased. The opposite trends in TS and EB could be explained by the polyhydroxy hydrophilic groups of glycerol, which can easily enter macromolecular chains in the film, decreasing molecular interactions between CNC and CS and the cohesion between polysaccharide chains. Thus, TS decreased [26]. Further, glycerol can increase the viscosity of the film solution, enhancing the shifting of molecular chains. This helps molecules extend during the film-forming process and increases the flexibility of prepared composite films, thereby increasing EB [27].

#### 2.2.4. Results of orthogonal array analyses

Single-factor tests showed that CNC, CS and glycerol addition had significant effects on the properties of the composite film. Based on the results of the single-factor experiments, the CNC/CS composite film with a closer thickness and better mechanical properties was selected to design an L_9_ (3^4^) orthogonal test, and the results are shown in table 2.

**Table 2.** Results of L_9_ (3^4^) orthogonal array design tests.

number	factor				results	
	A (%)	B (%)	C (%)	D	TS (MPa)	EB (%)
1	1 (0.75)	1 (1.00)	1 (0.50)	1	24.37	40.33
2	1	2 (1.50)	2 (0.75)	2	23.84	43.18
3	1	3 (2.00)	3 (1.00)	3	23.45	45.47
4	2 (1.00)	1	2	3	26.12	46.64
5	2	2	3	1	25.84	47.06
6	2	3	1	2	26.93	41.32
7	3 (1.25)	1	3	2	24.30	44.76
8	3	2	1	3	25.42	38.68
9	3	3	2	1	24.98	42.55
TS	K_1_	23.89	24.93	25.57		
	K_2_	26.30	25.03	24.98		
	K_3_	24.90	25.12	24.53		
	R	2.41	0.19	1.04		
EB	K_1_	42.99	43.91	40.11		
	K_2_	45.01	42.97	44.12		
	K_3_	42.00	43.11	45.76		
	R	3.01	0.94	5.65		

Through a stochastic analysis, factors affecting composite film TS were in the order A > C > B and the best plan was A_2_B_3_C_1_. Factors affecting composite film EB were in the order C > A > B and the best plan was A_2_B_1_C_3_.

As shown in table 3, ANOVA revealed that the concentration of CNC was a significant determinant of the composite film TS and EB. Glycerol concentration significantly affected the composite film TS and EB. The CS concentration did not significantly affect the mechanical properties of the composite film. Combining the results of ANOVA and stochastic analyses, the integrated plan was A_2_B_2_C_2_, where the CNC concentration was 1%, CS concentration was 1.5% and glycerol concentration was 0.75%. The CNC/CS composite film generated under these conditions had a thickness of 0.061 ± 0.0007 mm, TS of 27.21 ± 0.97 MPa and EB of 46.66 ± 1.05%, superior to that in previous studies [14].

**Table 3.** Variance analysis of orthogonal tests.

source	dependent variable	freedom	seq SS	adj SS	adj MS	F	*p*
A	TS	2	8.7856	8.7856	4.3928	1300.50	0.001**
	EB	2	14.107	14.107	7.053	36.01	0.027*
B	TS	2	0.0543	0.0543	0.0271	8.04	0.111
	EB	2	1.532	1.532	0.766	3.91	0.204
C	TS	2	1.6431	1.6431	0.8215	243.22	0.004**
	EB	2	50.757	50.757	25.378	129.56	0.008**
D	TS	2	0.0068	0.0068	0.0034		
	EB	2	0.392	0.392	0.196		
total	TS	8	10.4898				
	EB	8	66.787				

* P < 0.05, ** P < 0.01

### 2.3. Performance of composite films

#### 2.3.1. Morphology of composite films

**Figure 2.**
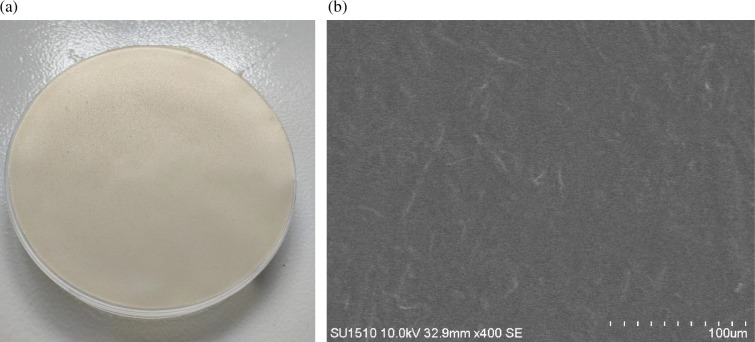
(a) Morphology of CNC/CS composite film; (b) SEM morphology of CNC/CS composite film.

As shown in figure 2a, the composite film was light yellow, similar to the colours of both CNC and CS, with a uniform thickness, no colour variation, a frosted, smooth and dense back surface, and no bubbles, scratches or stains. Figure 2b shows the microscopic morphology of the surface of the composite film. CNC was distributed more uniformly on the surface of the membrane, and part of the CNC clustered together to form protrusions, resulting in fine graininess on the membrane surface. The interior of the composite film was an interwoven mesh of long fibres.

#### 2.3.2. Water solubility and barrier properties of CNC/CS composite films

Water solubility (WS) is an important parameter for the practical application of thin films in environments with different moisture levels. As shown in table 4, the WS of the CS film was high, 32.36 ± 0.62%, due to abundant hydrophilic groups. The WS of the composite film was significantly lower (*p* < 0.05) at 16.88 ± 0.47% after the addition of nanocellulose. This was mainly due to the hydrogen bonding between the CNC and CS matrix, which occupied the hydroxyl group of the chitosan molecule, blocking water binding and reducing the WS of the composite film [28].

**Table 4.** Water solubility and barrier properties of CS and CNC/CS composite films.

film	WS/%	OP/×10^–4^ g/(m^2^﹒s)	WVP/×10^–10^ g/(m^2^·h·kPa)
CS	32.36 ± 0.62^a^	3.98 ± 0.27^a^	1.01 ± 0.16^a^
CNC/CS	16.88 ± 0.47^b^	2.12 ± 0.09^b^	0.68 ± 0.04^b^

Different letters in the same column indicate significant differences (p< 0.05).

Food packaging typically requires barrier properties to decrease the effects of the external environment (such as water, oxygen and UV light) [29]. The oxygen permeability (OP) value of the pure CS film was (3.98 ± 0.27) × 10^–4^ g/m^2^·s. After adding CNC, the OP value of the composite film decreased to (2.12 ± 0.09) × 10^–4^ g/m^2^·s (*p* < 0.05). Water vapour permeability (WVP) of the thin film reflects barrier properties and is usually influenced by environmental conditions, film structure and biopolymer properties. The WVP value of pure CS film was (1.01 ± 0.16) × 10^–10^ g/(m^2^·h·kPa). After adding CNC, the WVP value of the composite film was (0.68 ± 0.04) × 10^–10^ g/(m^2^·h·kPa), decreasing by 35.64% (*p* < 0.05). The barrier function of CNC in composite films can be explained by the Nielsen theory [30]. When the gas or liquid diffusing in the environment passes through composite films, it is blocked due to the presence of nanoparticles, decreasing the permeability [31]. This is consistent with the infrared spectroscopy results. Hydrogen bonding between CNC and CS decreases the number of hydrophilic groups in the system and the rate of water vapour transmission [32].

### 2.4. Characterization of bagasse cellulose and CNC/CS composite membranes

#### 2.4.1. Fourier transform infrared spectrometer (FT-IR) analysis

**Figure 3.**
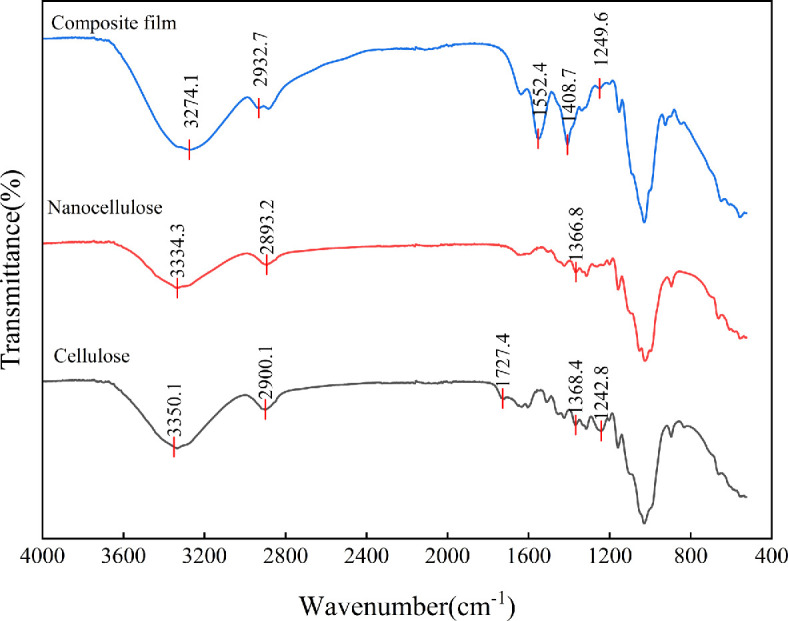
FT-IR spectra of cellulose, CNC and CNC/CS composite film.

Figure 3 shows the infrared spectra of cellulose, CNC and CNC/CS composite films. All samples exhibited obvious absorbance peaks around 3350 cm^−1^, which could be attributed to the O–H stretching vibrations in cellulose. Absorbance peaks around 2900 cm^−1^ were caused by the C–H stretching vibrations in cellulose. Absorbance peaks around 1727 cm^−1^ were caused by the C=O stretching vibrations in lignin and hemicellulose. Absorbance peaks around 1368 cm^−1^ were caused by bending vibrations of CH_2_ groups and C–H and C–O groups in aromatic rings in polysaccharides [33]. The absorbance peak at 1242 cm^−1^ was caused by vibrations of C–O bonds in lignin. In the CNC IR spectrum, the absorbance peaks at 1727 and 1242 cm^−1^ obviously decreased and even disappeared, suggesting that lignin and hemicellulose contents decreased significantly after alkali treatment. The absorbance peak of the composite film at 3274 cm^−1^ was a blue-shifted absorbance peak of cellulose, suggesting the presence of H bonds between cellulose and chitosan, enhancing the cohesion of composite films. Therefore, the mechanical properties of composite films were enhanced [34]. The absorbance peak at 2932 cm^−1^ was attributed to the antisymmetric and symmetric stretching vibrations of chitosan –CH_2_, respectively. The absorbance peak at 1552 cm^−1^ was due to the superposition of N–H bending vibration with the amide II vibration [24]. The absorbance peak at 1408 cm^−1^ became sharper, caused by the shear vibration of –NH_2_ in chitosan [35].

#### 2.4.2. X-ray diffraction(XRD) analysis

**Figure 4.**
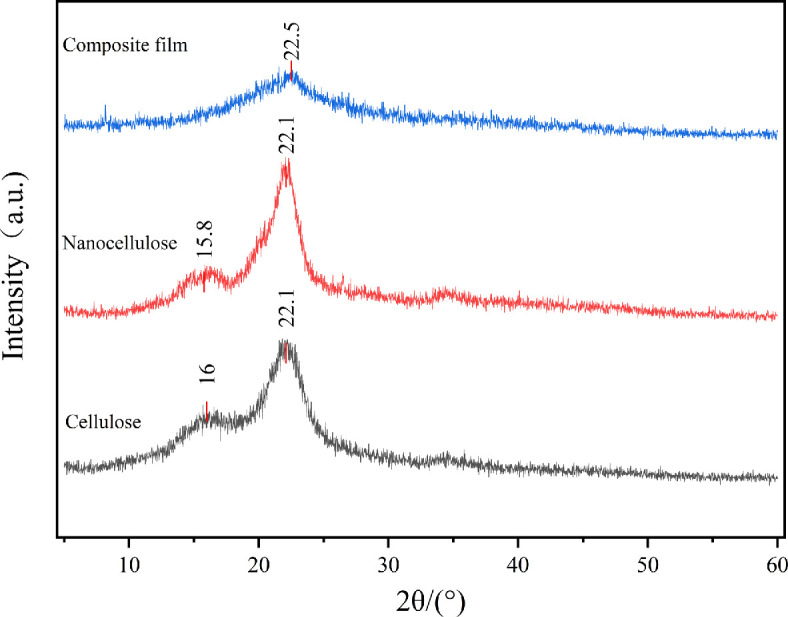
XRD patterns of cellulose, CNC and CNC/CS composite film.

Crystallinity is among the main factors affecting cellulose strength and mechanical properties. Figure 4 shows the XRD graphs of bagasse cellulose, nanocellulose and composite films. The main diffraction peaks of cellulose and nanocellulose were located at 16° and 22°, respectively, and these two diffraction peaks correspond to the {101} and {002} crystal planes of cellulose type I [36]. This result suggests that during nanosizing, the crystal type of cellulose did not change and still belonged to cellulose type I. Further observation of XRD patterns revealed a wider peak and lower intensity at 22.5°, suggesting that cellulose was already dispersed in the chitosan membrane matrix. This formed intense interactions with the thin film matrix, thus increasing the evenness of the composite film and decreasing the crystallinity [37]. The composite film did not show other peaks in the XRD pattern, suggesting that the addition of CS and glycerol did not alter the crystallinity of cellulose or induce the formation of new crystal types.

#### 2.4.3. Thermogravimetric analysis

**Figure 5.**
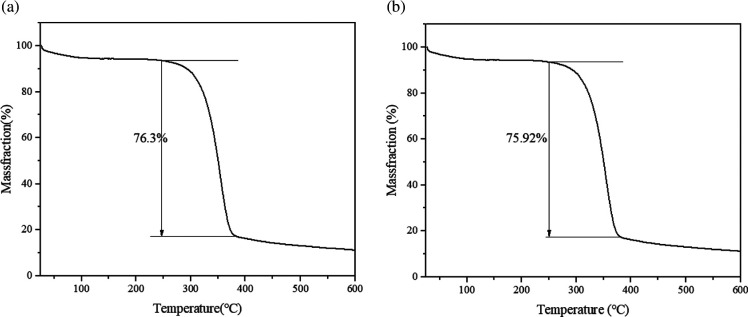
TG analysis of CNC (a) and CNC/CS composite film (b).

Thermogravimetric curves represent the inverse of the change in the weight loss rate during the warming process, as shown in figure 5 for the nanocellulose and CNC/CS composite membranes. The thermal degradation of the composite films and nanocellulose exhibited three main stages. Stage 1 occurred at 25−250°C, at which point the thermal weight loss rates of cellulose and composite membranes were low (approx. 4%) and the change was likely due to the removal of adsorbed or bound water in the samples by vapourization. Stage 2 occurred at 250–380°C; more mass was lost (approx. 76%), which could be attributed to the pyrolysis of the cellulose or membrane material itself, including depolymerization of the macromolecular chains, dehydration, decomposition of repeating units, oxidation and conversion of charred residues into gaseous low molecules. Stage 3 occurred between 380 and 600°C and mainly corresponded to the carbonization of remnants after degradation. The highest heat degradation temperatures of the composite film and CNC did not differ significantly.

## 3. Conclusion

Using bagasse as a raw material, cellulose was extracted by alkali treatment and nano-modified with concentrated sulfuric acid. Nanocellulose was successfully prepared with uniform particle sizes and high thermal stability. CNC/CS composite films were prepared using the newly prepared nanocellulose in CS films by mixing and extensional flow. The mechanical and barrier properties of composite films were significantly better than those of pure CS films. This is because nanocellulose and CS have H-bonding effects, forming a tight network structure and thus improving the mechanical and barrier properties of CNC/CS composite films. However, there were no changes in the crystal types and thermal stability was good. This study provides theoretical evidence for the high-value utilization of bagasse. CNC/CS composite films have broad applications in food preservation. Cellulose nanofibre-based composite membranes have excellent biocompatibility and high mechanical strength and can be used as substrates loaded with metal nanoparticles, natural antifungal compounds, etc., with a wide range of application prospects in food preservation. However, composite membranes may have problems such as not being able to balance barrier and antimicrobial properties, and the added antimicrobial preservatives generating microbial resistance, which need to be explored and solved by studying the composite membrane freshness preservation mechanism as well as the co-blend interaction mechanism.

## 4. Material and methods

### 4.1. Raw materials

The following materials were used: bagasse (Liuxing Sugar Manufacturing Co., Ltd., Liuzhou, China), chitosan (Shenzhen Shenbotai Biotechnology Co., Ltd., Shenzhen, China; deacetylation degree ≥95%, viscosity 100−200 mpa s), glycerol, sodium hydroxide and sulfuric acid (Chengdu Kelon Chemical Co., Ltd., Chengdu, China), glacial acetic acid (Chongqing Chuan Dong Chemical Co., Ltd., Chongqing, China) and sodium hypochlorite (Guangdong Wenglong Chemical Reagent Co., Shaoguan, China).

### 4.2. Preprocessing of bagasse

Bagasse was dried in an oven at 80°C for 6 h after cleaning, crushed and passed through a 60 mesh sieve. It was put aside for later use.

### 4.3. Preparation of bagasse CNC

Crushed bagasse was dispersed in a 30-fold volume of NaOH solution (10%) for 10 min, placed in a water bath at 80°C and reacted at a stirring rate of 500 rpm for 1 h. After centrifugation at 4000 r.p.m., the sample was poured into a NaClO solution and then oxidized and bleached for 40 min with the help of ultrasound (KQ-700DE; Kunshan Ultrasonic Instrument Co., Ltd., China) and effective chlorine (>10 g l^−1^). After the reaction was complete, the solution was centrifuged at 4000 r.p.m. and washed. The product was supplemented with 58% (mass fraction) sulfuric acid solution and stirred at 45°C at 400 r.p.m. for 1 h. Immediately after the completion of the reaction, a fivefold volume of distilled water was added to stop the reaction. Static stratification was used to obtain the suspension. After centrifugation at 7000 r.p.m. for 10 min, samples were rinsed with water thrice to remove impurities. The suspension (MD44-8000-14000; Hunan Yibo Biotechnology Co., Ltd., China) was dialyzed to neutral, ultrasonicated at 700 W for 20 min, dried in a vacuum and pulverized to obtain CNC. The yield of CNC, *y* (%), is calculated using equation (4.1).


(4.1) $$y=\frac{m}{m_{0}}\times 100,$$

where *m* is the mass of cellulose after nanosizing and *m*_0_ is the mass of the original cellulose.

### 4.4. CNC/CS composite film process optimization

#### 4.4.1. Preparation of the CNC/CS composite film

The prepared nanocellulose was added to an appropriate amount of distilled water, followed by ultrasonication at 700 W for 20 min for dispersion. CS was dissolved in an acetic acid (2% v/v) solution, and then the CS solution and glycerol were added to the nanocellulose solution. The sample was stirred for 10 min, followed by 500 W ultrasonic defoaming for 10 min and then casting to form a film on a glass plate. The sample was then removed from the mold by drying for 8 h at 55°C. The prepared composite film was equilibrated in a constant temperature and humidity chamber at 25°C and 60% relative humidity for 24 h.

#### 4.4.2. Single-factor experiments

(1) Influence of different CNC concentrations on the performance of the composite film

Using a CS concentration of 1% and glycerol concentration of 1%, CNC was added at 0.5, 0.75, 1, 1.25 and 1.5%. The effects of various CNC concentrations on the composite film thickness, TS, and EB were observed.

(2) Influence of different CS concentrations on the performance of the composite film

Setting the CNC concentration to 1% and glycerol concentration to 1%, CS was added at 0.5, 0.75, 1, 1.25 and 1.5%. The effects of various CS concentrations on the composite film thickness, TS and EB were observed.

(3) Influence of different glycerol concentrations on the performance of the composite film

Setting the CNC concentration to 1% and CS concentration to 1%, glycerol was added at 0.5, 0.75, 1, 1.25 and 1.5%. The effects of various glycerol concentrations on the composite film thickness, TS, and EB were observed.

#### 4.4.3. Orthogonal design

Based on the single-factor experiment results, an orthogonal experimental design L_9_ (3^4^) was performed to obtain the optimal properties of the complex film using the orthogonal design software (Minitab 15). The four processing factors included CNC concentration (A), CS concentration (B), glycerol concentration (C) and blank (D). The three levels of each factor are shown in table 2. Mechanical properties (TS and EB) were indicators of the characteristics of the composite films.

### 4.5. Performance of bagasse nanofibs and composite films

#### 4.5.1. Particle size determination

The prepared CNC was dispersed using water and a nanoparticle system (Nano ZS90; UK Malvern Panalytical Ltd.) and used for grain size analyses.

#### 4.5.2. Determination of film thickness

A smooth, flat CNC/CS composite film was selected, and the thickness was measured at the centre of the film as well as at six flat planes around the film using a spiral micrometer (IP64; SANRYO, Japan); the average value was recorded.

#### 4.5.3. Tensile strength and elongation at break

TS and EB of the membranes were tested using a digital push/pull force gauge meter (HLD; Yueqing Aidelberg Instrument Co., Ltd., China). Clean, even samples were selected and cut into 1.5 mm × 50 mm rectangles. The initial pulling force was 0.5 N. The samples were stretched at a constant pace until the film was broken. The stretching length (mm) and pulling force (N) at the time were recorded. TS (MPa) and EB (%) are calculated using equations (4.2) and (4.3).


(4.2) $$TS=\frac{F}{S}$$

(4.3) $$EB=\frac{L_{1}-L_{0}}{L_{0}}\times 100,$$

where *F* is the maximum tensile force (N) when the film breaks, *S* is the cross-sectional area of the film, *L*_1_ is the length of the film when it breaks (mm) and *L*_0_ is the initial length of the film sample (mm).

#### 4.5.4. Water solubility

CNC/CS composite films were cut into 2 cm × 2 cm pieces, dried at 105°C for 24 h, and weighed, and this weight was recorded as *m*_1_. Subsequently, dried composite films were soaked in 30 ml of ultrapure water and incubated at 25℃ for 24 h. The thin film was dried at 105°C again for 24 h, and this weight was recorded as *m*_2_. WS (%) is calculated using equation (4.4) [38].


(4.4) $$WS=\frac{m_{2}-m_{1}}{m_{1}}\times 100.$$

#### 4.5.5. Water vapour permeability and oxygen permeability

Following the ASTME96/E96M-10 standard testing method, WVP was measured using the desiccant method [39]. Briefly, 15 g of silica was added to the weighing cup, and the relative humidity in the cup was kept at 0%. A specimen with a diameter slightly larger than that of the weighing cup was then sealed above the weighing cup. The weighing cup was put in an environment of 25°C and 60% relative humidity. Changes in the mass of the weighing cup were recorded. WVP is calculated using equation (4.5) (10^−4^ g/(m·h·kPa)).


(4.5) $$WVP=\frac{\Delta G\times d}{t\times A\times \Delta P},$$

where Δ*G* is the mass change of the weighing cup (g), *d* is the thickness of the composite membrane (m), *A* is the effective area of the composite membrane (m^2^), *t* is the interval time (h) and Δ*P* is the pressure difference between the two sides of the composite membrane (kPa).

Based on the oxidation principle of iron, OP was measured using deoxidizer adsorption [40]. Briefly, 1.5 g of sodium chloride, 1 g of activated carbon and 0.5 g of reduced iron were placed in the bottom of a conical flask. The bottleneck was sealed using a thin film. It was put at the bottom of the drying dish with saturated barium chloride solution and weighed every 24 h for 7 days. OP (10^−4^ g/m^2^·s) is calculated using equation (4.6).


(4.6) $$\text{OP}=\frac{m-m_{0}}{t\times s}.$$

In the formula, *m* is the weight of the weighing bottle (g), *m*_0_ is the initial weight of the weighing bottle (g), *t* is the interval time (h) and *s* is the area of the permeable surface (m^2^).

#### 4.5.6. SEM analysis

Cellulose and CNC were sampled using monocrystalline silicon wafers. The CNC/CS composite film was cut into 2 mm × 2 mm samples fixed on conductive adhesive tapes, sprayed with gold and observed using a scanning electron microscope (SU1510; Hitachi, Japan) with an accelerating voltage of 10 kV.

#### 4.5.7. Fourier transform infrared spectrometer analysis

Bagasse, CNC and CNC/CS composite films were 55% lyophilized and crushed into powder. The powder was homogeneously dispersed in KBr, pressed and characterized using a Fourier transform infrared spectrometer (IRPrestige-21; Shimadzu, Kyoto, Japan) at a wavelength of 4000−400 cm^−1^.

#### 4.5.8. X-ray diffraction analysis

Bagasse cellulose, CNC and CNC/CS composite membranes were analysed for crystalline properties by an X-ray diffractometer (XRD-6100; Shimadzu, Japan) with a scanning range of 5°−60°, scanning speed of 8°/min, and operating conditions of 40 kV and 20 mA.

#### 4.5.9. Thermogravimetric analysis

CNC and CNC/CS were ground into a powder and analysed using a thermogravimetric analyser (STA4493F3; NETZSCH Instruments GmbH, Germany) with nitrogen as the protective gas at a flow rate of 20 ml min^−1^, temperature increase rate of 20°C min^−1^ and temperature increase range of 25−600°C.

### 4.6. Statistical analyses

Each experiment was repeated three times. The results are presented as the mean ± standard deviation. Experimental data were analysed using SPSS 19.0 and graphs were plotted using Origin 9.1.

## Data Availability

Data provided as supplementary material [41]. Supplementary material is available online [42].
